# Detecting focal cortical dysplasia lesions from FLAIR-negative images based on cortical thickness

**DOI:** 10.1186/s12938-020-0757-8

**Published:** 2020-02-22

**Authors:** Cuixia Feng, Hulin Zhao, Maoyu Tian, Miaomiao Lu, Junhai Wen

**Affiliations:** 1 Department of Biomedical Engineering, School of Life Science, Beijing Institute of Technology, Beijing, China; 2 Sixth Medical Center of PLA General Hospital, Beijing, China

**Keywords:** Cortical thickness, Epilepsy, FCD, FLAIR-negative image

## Abstract

**Background:**

Focal cortical dysplasia (FCD) is a neuronal migration disorder and is a major cause of drug-resistant epilepsy. However, many focal abnormalities remain undetected during routine visual inspection, and many patients with histologically confirmed FCD have normal fluid-attenuated inversion recovery (FLAIR-negative) images. The aim of this study was to quantitatively evaluate the changes in cortical thickness with magnetic resonance (MR) imaging of patients to identify FCD lesions from FLAIR-negative images.

**Methods:**

We first used the three-dimensional (3D) Laplace method to calculate the cortical thickness for individuals and obtained the cortical thickness mean image and cortical thickness standard deviation (SD) image based on all 32 healthy controls. Then, a cortical thickness extension map was computed by subtracting the cortical thickness mean image from the cortical thickness image of each patient and dividing the result by the cortical thickness SD image. Finally, clusters of voxels larger than three were defined as the FCD lesion area from the cortical thickness extension map.

**Results:**

The results showed that three of the four lesions that occurred in non-temporal areas were detected in three patients, but the detection failed in three patients with lesions that occurred in the temporal area. The quantitative analysis of the detected lesions in voxel-wise on images revealed the following: specificity (99.78%), accuracy (99.76%), recall (67.45%), precision (20.42%), Dice coefficient (30.01%), Youden index (67.23%) and area under the curve (AUC) (83.62%).

**Conclusion:**

Our studies demonstrate an effective method to localize lesions in non-temporal lobe regions. This novel method automatically detected FCD lesions using only FLAIR-negative images from patients and was based only on cortical thickness feature. The method is noninvasive and more effective than a visual analysis for helping doctors make a diagnosis.

## Background

Focal cortical dysplasia (FCD) is a malformation of cortical development (MCD) and is also one of the most common causes of intractable epilepsy that was defined by Taylor et al. in 1971 [1]. In the clinical treatment of drug-resistant epilepsy, surgical resection is often used to remove the lesion area. Automated techniques for FCD detection can be of great assistance to the neuroradiologist. Therefore, the localization of epileptogenic foci before surgery plays an important role in the diagnosis, surgical evaluation, and prognosis of epilepsy.

Several conventional methods for the detection of epileptic foci include the voxel-based morphometry algorithm (VBM) [2–5], the surface-based morphometry algorithm (SBM) [6, 7] and the postprocessing method [8, 9] based on voxel feature extraction. The VBM technique is mainly based on the image density, compared with the normal template, and the abnormal area found in the image is taken as the lesion area. The SBM technique is mainly used to reconstruct the cerebral cortex, extract effective features, and use a machine learning method to classify and find the lesion area. The postprocessing method is used to extract features, such as texture features and cortical thickness features, construct a computational model, and find the location of the lesion. At present, there are two novel studies examining FCD lesion recognition and location using an advanced convolutional neural network (CNN), which have achieved effective results [10, 11].

Magnetic resonance imaging (MRI) is noninvasive and effective in the diagnosis and evaluation of epileptogenic foci before an operation. MRI features of FCD show focal cortical thickening, fuzziness between grey matter (GM) and white matter (WM), cortical/subcortical WM hyperintensity on T2-weighted imaging (T2WI)/fluid-attenuated inversion recovery (FLAIR), widened gyri and abnormal sulci [12]. Cortical thickness is a kind of distance measurement between the inner and outer surfaces of the GM, and it is an important morphological index of the cerebral cortex. Among the MRI features, the increase in cortical thickness is the most obvious feature of cortical dysplasia, especially FCDs [13]. Studies have shown that increased cortical thickness occurs in 91% of patients [14]. The epileptogenic areas induced by FCD often show high signal and cortical thickening or brain volume abnormalities [15] (atrophy or hypertrophy) on FLAIR images.

Cortical thickness is a precise and reliable measurement for subtle and focal changes in MR images, and it has mainly been used as the feature in the image to identify the lesion area. In 2001, Bernasconi used run-length coding (RLC) to measure the thickness of GM as a feature to detect FCD lesions [8]. When using RLC to calculate the midline area of the brain, especially the cingulate gyrus, it is easy to make many errors. Antel used Jones’ Laplace method to calculate the cortical thickness in the image as the feature, which was better than RLC in the recognition of lesions [9]. The cortical thickness measured by the automated segmentation with proximities (ASP)/constrained Laplacian-based ASP (CLASP) methods has been used to classify schizophrenic patients and normal people. The results showed that cortical thickness is a reliable quantitative feature for pattern classification [16]. Alzheimer’s disease patients and normal people have also been studied through cortical thickness [17]. In a recent study, using the cortical thickness extracted based on SBM as a feature, this method determined 92% of cortical lesions (sensitivity) and few false positives (96% specificity), thereby successfully distinguishing patients from normal people [13].

At present, there are two main methods to measure the cortical thickness of the cortex. One is based on VBM, which depends solely on image intensities to determine the inner and outer surface of the cortex and then calculates the thickness of the cortex [18, 19]. The other is based on SBM, which deforms the inner and outer cortical surfaces and then calculates the thickness between these cortical surfaces [20, 21]. Other common methods for cortical thickness measurement include projection-based thickness (PBT) [22], minimum line integrals on soft-classified tissue [23] based on minimizing line integrals over the probability map of the GM in the MRI volume, voxel-based cortical thickness (VBCT) maps [24], and measurement of cortical thickness based on differential homeomorphism [25]. The most common cortical thickness measurement tools are Automatic Regional Cortical ThICkness (ARCTIC) [26, 27] a plug-in for 3D Slicer, Civet-CLASP [28, 29], and FreeSurfer [20]. Using VBM to calculate the cortical thickness is fast, it does not need to build an accurate surface topology structure, and it is more convenient to combine with other images to identify the lesion area. A study examined the precision of cortical thickness measurements and compared six different cortical thickness metrics, showing that the Laplace metric precisely measures cortical thickness [30]. Additional research has indicated that Laplace is effective [31].

Although these currently available techniques for the detection of FCD lesions have shown good performance, these techniques are sensitive to artefacts and have high computational complexity. In addition, the current research has mainly focused on either T1-weighted images (T1WI) or T2WI, or researchers have combined FLAIR images with T1WI or T2WI to study epilepsy. Although T1W images are superior for measuring cortical thickness and identifying the interface between GM and WM, FLAIR images are more sensitive in detecting cortical and subcortical hyperintensities than T1WI and are more sensitive in identifying subtle lesions. The increase in cortical thickness is the most obvious feature of cortical dysplasia, and an assessment of increased cortical thickness can provide valuable information for lesion location. Therefore, we consider using the Laplace method to calculate the cortical thickness as an important feature to identify lesions in FLAIR-negative images. We are working towards a novel automated method that is able to effectively detect FCD lesions automatically from FLAIR-negative images based on comparison between the cortical thickness of the individual and that of the normal template. This technique is focused on improving the visualization of lesions based only on the cortical thickness in FLAIR-negative images. It is meaningful for doctors to improve the detection of FCD lesion areas in epileptic patients who were pathologically confirmed to have FCD lesions but with normal FLAIR images (herein called FLAIR-negative images) instead of FLAIR-positive images.

## Results

**Fig. 1:**
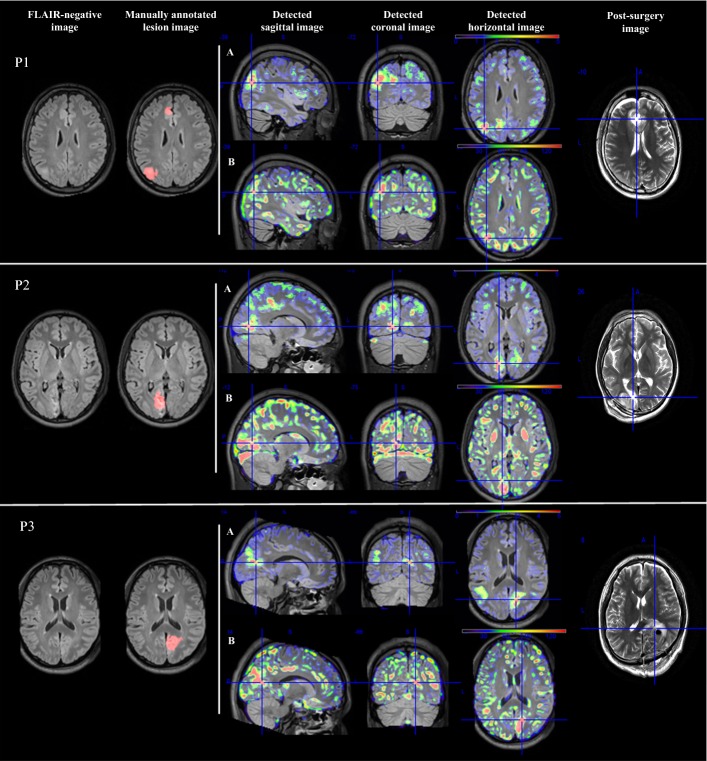
Detection results with three patients. The first column is the original image. The second column is the image of the manually marked lesion area. The third to fifth columns are the detection results in the sagittal plane, coronal plane and horizontal plane, respectively, and the cross-hairs indicate the recognized position. In these columns, row A represents the results of the proposed method, while row B represents the extension map detected by MAP. The sixth column is the image of the postoperative image, and the cross-hairs indicate the position after the operation

Three of the four lesions in three patients with non-temporal lobe epilepsy were detected. The detection results in the three patients are shown in row A in Fig. 1. We also used an extension map based on GM density in the morphometric analysis program (MAP) algorithm [5] to locate the lesion area, as shown in row B in Fig. 1.

For the detected image, if the value of the cortical thickness extension map was greater than three, we considered the voxel as a lesion voxel. The quantitative evaluation of the three patients with the detected lesion is shown in Table 1.

**Table 1:** Quantitative results of the detected lesions (%)

Metrics	P1	P2	P3	Mean ± SD
Specificity	99.86	99.54	99.94	99.78 ± 0.21
Accuracy	99.85	99.52	99.93	99.76 ± 0.21
Recall	74.34	67.71	60.32	67.45 ± 7.01
Precision	18	11.13	32.13	20.42 ± 10.70
Dice coefficient	28.98	19.12	41.93	30.01 ± 11.43
Youden index	74.2	67.25	60.26	67.23 ± 6.97
AUC	87.1	83.63	80.13	83.62 ± 3.48

We quantitatively analysed the comparison between the proposed method and the existing techniques, as shown in Table 2. The detection results of the FLAIR-negative image in this study using the MAP algorithm are shown in row five of Table 2.

**Table 2:** Performance comparison with existing techniques

Related work	Method	Data type	Patient-wise	Voxel-wise		
			Recall	Recall	Precision	Dice coefficient
Wong-Kisiel et al. [4]	VBM	T1-weighted	64	–	–	–
Ahmed et al. [6]	SBM	T1-positive	85	20.14	–	22.36
		T1-negative	58	2.47	–	3.68
Bijay Dev et al. [10]	CNN	FLAIR	82.5	40.1	80.69	52.47
Wagner et al. [5]	VBM	FLAIR-negative images from this study were used	50	19.47	10.27	13.36
*Proposed method*	*VBM*	*FLAIR*-*negative*	*50*	*67.45*	*20.42*	*30.01*

We counted FCD lesion volumes, which were manually segmented, and these values ranged from 1545.75 to 9412.88 mm^3^. We divided the patients into two groups: one group was those with non-temporal lobe epilepsy (non-TLE), which included epileptic patients whose lesions occurred in an area outside the temporal lobe, and the other group was those with temporal lobe epilepsy (TLE), which included epileptic patients whose lesions occurred in the area of the temporal lobe. We calculated the average cortical thickness in different regions in different images. The comparison between the cortical thickness of the lesion area of the patient and that of the healthy controls (HCs) is shown in Table 3. The FCD lesion refers to the manually segmented lesion area. The contralateral area of the FCD was the symmetrical area of the midsagittal plane (MSP) of the brain in this patient. The corresponding area of the FCD in the mean image and standard deviation (SD) image refers to the corresponding regions in the patient in the manually segmented FCD region in the HCs.

**Table 3:** The statistical average cortical thickness in different regions

Type	P	Patients		Healthy controls	
		FCD area	Contralateral area of FCD	Corresponding area of FCD in mean image	Corresponding area of FCD in SD image
Non-TLE	P1	10.80	6.14	2.77	2.54
		*10.12*	*7.90*	*6.03*	*3.30*
	P2	6.28	4.84	3.01	2.63
	P3	7.73	4.04	3.57	2.51
*Mean ± SD*		*8.73 ± 2.10*	*5.73 ± 1.69*	*3.84 ± 1.49*	*2.75 ± 0.37*
TLE	P4	8.24	8.21	9.65	2.68
	P5	9.22	8.69	9.87	2.90
	P6	9.55	10.16	9.82	2.79
*Mean ± SD*		*9.00 ± 0.68*	*9.02 ± 1.02*	*9.78 ± 0.12*	*2.79 ± 0.11*

We also calculated the mean and SD of different anatomical areas of the cortical thickness mean image according to the labels provided by Neuromorphometrics. Table 4 shows some selected areas with a mean cortical thickness greater than 4.5 mm. The label numbers in the first column and the names of the anatomical area in the second column were defined by the labels provided by Neuromorphometrics.

**Table 4:** Mean and SD cortical thickness (in mm) for thicknesses greater than 4.5 mm

Label	Anatomical area	Cortical thickness (right/left) (mm)	
		Mean	SD
23/30	Accumbens area	9.98/10.47	1.77/1.99
31/32	Amygdala	12.51/12.56	2.50/2.28
36/37	Caudate	5.89/5.97	2.49/2.47
47/48	Hippocampus	7.71/7.78	2.43/2.34
57/58	Putamen	11.52/11.76	4.20/4.46
75/76	Basal forebrain	5.91/5.79	2.99/3.02
*100/101*	*ACgG anterior cingulate gyrus*	*5.85/5.88*	*2.91/2.93*
102/103	AIns anterior insula	6.83/6.77	3.12/3.12
104/105	AOrG anterior orbital gyrus	4.63/4.71	2.64/2.53
116/117	Ent entorhinal area	9/9.13	2.91/3.22
118/119	FO frontal operculum	4.71/4.65	3.03/2.84
122/123	FuG fusiform gyrus	6.07/6.44	3.24/3.31
132/133	ITG inferior temporal gyrus	5.48/5.62	3.37/3.47
138/139	MCgG middle cingulate gyrus	4.56/4.49	2.77/2.79
140/141	MFC medial frontal cortex	5.47/5.35	2.76/2.73
146/147	MOrG medial orbital gyrus	4.80/4.54	2.61/2.58
154/155	MTG middle temporal gyrus	5.43/5.26	3.47/3.44
170/171	PHG parahippocampal gyrus	4.75/4.49	2.35/2.40
172/173	PIns posterior insula	5.64/5.67	2.62/2.77
178/179	POrG posterior orbital gyrus	5.01/4.92	2.68/2.71
186/187	SCA subcallosal area	5.00/5.16	2.60/2.56
200/201	STG superior temporal gyrus	4.81/4.68	3.37/3.34
202/203	TMP temporal pole	5.32/5.31	2.87/2.88

**Fig. 2:**
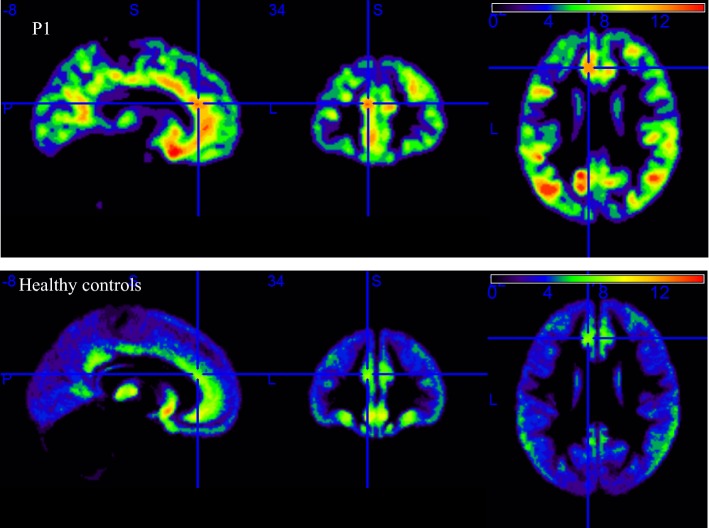
The first row is the cortical thickness image for patient P1, and the cross-hairs indicate the actual location of the lesion. The second row shows the cortical thickness features of healthy controls

Patient P1 had two lesions. One of the lesions was detected, as shown in row A in Fig. 1, and the other was missed, as shown in Fig. 2.

## Discussion

This method is effective in detecting epilepsy in non-temporal lobe areas. Three of the four lesions in the non-temporal areas of FLAIR-negative images from three patients were detected, as shown in Fig. 1. We observed that the cortical thickness extension maps showed an increase in the lesion area. At present, many algorithms can find FCD II lesions in MR images, but it is difficult to detect FCD I lesions. In this study, for patients P1 and P2 with FCD Ib lesions, the localization results were better. However, no lesions were detected for TLE.

Comparing the detection results of the proposed method and MAP [5] in Fig. 1 and Table 2, we can conclude that the proposed method was better. Although the MAP method can also detect FCD lesions, there were too many false-positive areas detected. This is because the MAP method is mainly based on the density of images, where the extension map of MAP identifies abnormal extension of GM into WM and the junction map of MAP is sensitive to the blurry area at the GM-WM junction. However, the densities of GM and WM in the FLAIR image are very close, and the boundary is very unclear.

The voxel-wise analysis of the results of the quantitative detection of lesions with the proposed method is reported in Table 1, in which the specificity and accuracy are relatively high, mainly due to class imbalance in that a large number of voxels belong to healthy voxels. The recall was 67.45%, which indicated the percentage of voxels in the manual segmentation label that were classified as a lesion voxel. It can be seen that a large number of lesion voxels were recognized. The Dice coefficient was 30.01%, which reflects the correlation accuracy of the segmentation, and this method balances recall and precision. In addition, the area under the receiver operating characteristic (ROC) curve (AUC) was 83.62%, which indicated a good classification performance of the proposed method.

Table 2 shows the performance comparison with existing techniques and provides some results with VBM, SBM, and CNN. The current VBM method is mainly to enhance the FCD lesion area, and its results are mainly patient-wise, and therefore lacks voxel-wise measurement results. Ahmed et al. used the SBM method to analyse T1-positive and T1-negative images, and T1-positive images had good results. Bijay Dev et al. first used a CNN model to automatically segment FCD lesions with only FLAIR images. This method achieved the best segmentation results, mainly because the purpose of the study was to perform segmentation, there was no mention of whether the FLAIR image was positive or negative. Indeed, CNN technology has achieved good results in the field of image recognition and location. The comparison of two VBM techniques for the detection of FLAIR-negative images also showed that the proposed technique can provide higher detection and segmentation accuracy.

### Detection of epilepsy in the temporal lobe

Previous studies have shown that the cortical thickness in the human brain varies considerably, from approximately 2 mm in the calcarine sulcus to approximately 4 mm in the precentral gyrus, with an average of approximately 3 mm [32–35]. In this study, the average cortical thickness of the different anatomical areas of HCs was calculated according to the labels provided by Neuromorphometrics. The thickness of most areas was in the range of 2 to 4 mm, which was consistent with previous studies.

The detection results of lesions that occurred in the non-temporal areas were positive, but for the three patients whose lesions occurred in the temporal lobe area, the detection results were not ideal. In Table 3, the average cortical thickness in those with non-TLE lesions was larger than that in the HCs, but the difference in average cortical thickness was very small between the HCs and the patients with TLE lesions. For example, the average cortical thickness of the lesions in patient P6 was 9.55 and the average cortical thickness in HCs was 9.82. They were very similar, and the final z-score values were very small, so it was very difficult to accurately locate the FCD lesions in the temporal lobe based only on cortical thickness. Table 4 shows that the cortical thickness of the anatomical structural cortex near the temporal lobe on the left and right sides was relatively thick. This in itself increased the difficulty for localizing lesions in the region.

### Patient P1 had two lesions

Patient P1 had two lesions, one of which was detected and the other was missed. However, the missed lesion was confirmed as actual epileptic foci by cortical electroencephalogram (ECoG). Table 4 shows that the missed lesion in patient P1 was located in the anterior cingulate gyrus. The mean cortical thicknesses of the left and right sides for this area were 5.85 and 5.88, respectively, and the SDs were 2.91 and 2.93, respectively. As shown in Table 3, the average cortical thickness of the lesion not detected in patient P1 was 10.12 mm, and the average cortical thickness in HCs in this area was 6.03, and the SD was 3.30. The z-score value of the lesion not detected in patient P1 was 1.24, and because the threshold was three, this lesion was ignored. The smooth cortical thickness for patient P1 and the cortical thickness mean image for HCs are shown in Fig. 2. The area in the normal template image corresponding to patient P1’s undetected lesion also had a slightly thick cortex, as shown in the second row of Fig. 2. Therefore, if the cortex of the patient and that in the normal template in a certain area are both relatively thick, the lesion area may be missed during the comparison process.

### Limitations

The accuracy of the location in patient P2 was slightly lower, which was mainly caused by the GM segmentation. Because the boundary of the GM and WM in the FLAIR image was not clear, the detection accuracy for lesions depends on the correct segmentation of the cortex. At the same time, the lesions marked by doctors by hand may not be particularly accurate, which may have reduced the performance of the quantitative analysis. Therefore, the subsequent GM segmentation and manual labelling of lesions need to be further optimized.

Although our recognition results are good, there were still false-positive areas. If the threshold of the cortical thickness extension map was reduced from three to two, all the lesions can be identified, but the number of false-positive areas would increase. The follow-up study can use the present method in combination with other effective features to locate the lesion area and remove more false-positive areas.

## Conclusion

We presented a normalized mean cortical thickness map of the brain, and then based on this map, we located abnormal cortical areas of the brain from FLAIR-negative images. FCD lesions can be found by comparing cortical thickness in patients to normal controls. This method was effective for locating the lesions in the non-temporal lobe areas. This method can be used not only for any modal image, but also for any disease related to the thickness of the cortex, as long as the GM and WM can be accurately segmented.

Noninvasive measurement and analysis of cortical thickness based on MR images is of great significance for the study of disease occurrence and has become an important research method in brain science. Abnormal cortical thickening or thinning is related to neurological diseases. Cortical thickening occurs in epileptic patients with cortical dysplasia, while abnormal cortical thinning occurs in patients Alzheimer’s disease (AD) or schizophrenia. Therefore, cortical thickness as a feature can provide effective information about normal or abnormal areas. The measurement of cortical thickness can be applied to disease monitoring and in research.

## Materials and methods

### Materials

#### Patients

Six patients were selected (average age ± SD = 32 ± 13; four males and two females) from the Sixth Medical Center of PLA General Hospital (Haidian District, Beijing, China) between 2012 and 2016 who had three-dimensional (3D) high-resolution FLAIR-negative images. The inclusion criteria included the following: (1) all patients underwent surgical resection of the FCD lesions to treat drug-resistant epilepsy, and epilepsy did not reoccur. (2) All patients were confirmed with histological FCD based on classification standards [32, 36]. (3) All patients had preoperative 3T high-resolution FLAIR images that were negative. A total of seven lesions were detected in the six patients and patient P1 had two lesions. Pathological reports based on resected tissues confirmed that three patients were FCD I b, one was FCD II a and two were FCD II b. The surgery was based on strong clinical and electroencephalogram (EEG) localizing information. The manual segmentation of the FCD lesion area was performed by an epilepsy expert with 18 years of clinical experience. The detailed patient demographics are shown in Table 5.

**Table 5:** Detailed patient demographics and FCD information

No.	Year/onset age	Sex	Surgical resection region	FCD type
P1	1993/23	Male	Left frontal lobe, cingulate gyrus	I b
P2	2003/14	Male	Left occipital lobe	I b
P3	1986/29	Male	Right occipital lobe	II b
P4	1970/46	Male	Right temporal lobe	I b
P5	1984/32	Female	Left temporal lobe	II a
P6	1971/45	Female	Left temporal lobe	II b

#### MRI acquisition

Preoperative 3D MR images were acquired on a 3T scanner (SIEMENS Skyra) using a FLAIR sequence (repetition time (TR) = 5000 ms, echo time (TE) = 396 ms, flip angle = 120°, slice thickness = 0.4688 mm, displayed field of view (DFOV) = 195 mm) with an isotropic voxel size of 0.4688 *0.4688 *0.4688 mm and the size of the images was 320 * 416 * 512. Approval for the study was obtained from the Sixth Medical Center of PLA General Hospital institutional ethics committee.

#### Healthy controls (HC) or normal subject database

All HCs come from the IXI (Information eXtraction from Images) Dataset (http://brain-development.org/ixi-dataset/). The dataset includes nearly 600 MR images from normal, healthy subjects. The MR image acquisition protocol for each subject included T1-, T2- and proton density (PD)-weighted images, magnetic resonance angiography (MRA) images and diffusion-weighted images (15 directions). We used T1 images from 32 age-matched subjects from Hammersmith Hospital using a Philips 3T system. The details of the scanner parameters are as follows: TR = 9.6, TE = 4.60, number of phase encoding steps = 208, echo train length = 208, reconstruction diameter = 240.0, acquisition matrix = 208 × 208, and flip angle = 8.0°.

#### Image processing

All digital imaging and communication (DICOM) data from the MR scanner were converted into 3D images in the Neuroimaging Informatics Technology Initiative (NIfTI) format by dcm2nii software. All images were analysed on a Dell computer (Intel(R) Xeon(R) CPU E5-1607 v3 @3.10 GHz, 4 GB RAM) using Statistical Parametric Mapping 12 (SPM) [33]. We used the labels_Neuromorphometrics.nii from SPM12 to remove the cerebellum area. The neuromorphometric also called maximum probability tissue labels derived from the ‘MICCAI 2012 Grand Challenge and Workshop on Multi-Atlas Labelling’ (https://masi.vuse.vanderbilt.edu/workshop2012/index.php/Challenge_Details). The labelled data (labels_Neuromorphometrics.nii) were provided by Neuromorphometrics, Inc. (http://Neuromorphometrics.com/). These data were released under the Creative Commons Attribution-NonCommercial (CCBY-NC) licence with no end date.

## Methods

**Fig. 3:**
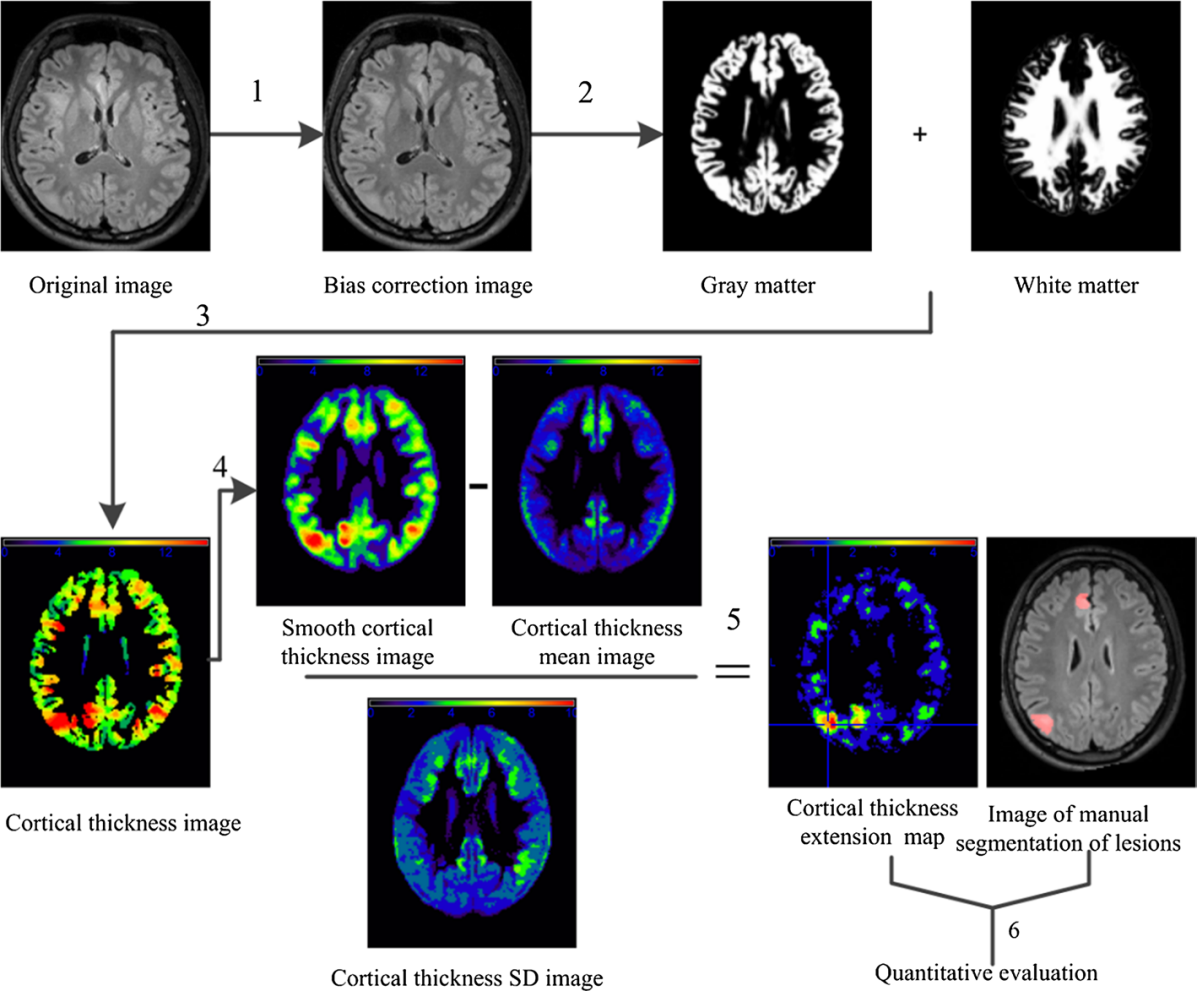
Overview of the image processing steps for identifying lesions based on cortical thickness: (1) bias correction, (2) normalized segmentation, (3) cortical thickness calculation, (4) convolution, (5) comparison with healthy controls (see text for details), and (6) quantitative evaluation

The methodology used in this study consisted mainly of preprocessing, constructing a cortical thickness extension map and quantitative evaluation, and the detailed process mainly included six steps: (1) bias correction, (2) normalized segmentation, (3) cortical thickness calculation, (4) convolution/smoothing, (5) comparison with HCs, and (6) quantitative evaluation. The preprocessing included the first step and the second step, mainly using SPM12. Constructing a cortical thickness extension map comprised the third step to the fifth step. The 3D Laplace method was used to calculate the thickness of the cortex in step three. In the fourth to sixth steps, MATLAB was used to realize the detection and evaluate the differences in the lesion area between the detection results and manually segmented results. The overall structural flow chart is shown in Fig. 3.

The main steps are as follows:

1. Bias correction: bias correction corrected the density of the image and removed the bias artefacts due to the physics of MR scanning, which is a process that makes the image more conducive to automatic processing. The bias-corrected version should have more uniform intensities within the different types of tissues. The parameters in SPM12 were as follows: bias regularization = light regularization (0.001); Bias FWHM = 60 mm.
2. Normalized segmentation: the individual subject images were normalized by performing a nonlinear deformation field based on the tissue probability maps (TPM) through affine registration. Meanwhile, the normalized image was segmented into six different tissue types, including the GM, WM, cerebrospinal fluid (CSF), bone, soft tissue, and air/background, using TPM. The parameters in SPM12 were as follows: tissue probability map = TPM; The number of Gaussians is set 1, 1, 2, 3, 4, 2 by default; Markov Random Field (MRF) parameter = 1; Clean up = light clean; Warp regularization = [0 0.001 0.5 0.05 0.2]; Affine regularization = east Asian brains; Smoothness = 0; Sampling distance = 3.
3. Cortical thickness calculations: measuring the cortical thickness required two major processing steps: cortical segmentation and thickness calculation. This cortical segmentation, which is done in step 2, yielded two surfaces: the WM–GM surface between WM and GM and the GM-CSF surface between GM and CSF. The calculation of cortical thickness involved calculating the distance between the two surfaces. The Laplace method was used to calculate the thickness of the GM cortex in 3D images and included three steps: first, the initial equipotential surface through the inner and outer boundaries of the cerebral cortex were defined, each equipotential surface between the two boundaries were iteratively solved, then the normal vector of the equipotential surface was calculated, and finally, the thickness of the cortex was calculated by the field line tracking method. The detailed process of these three steps was as follows:
  Step 1: the GM-CSF surface and WM–GM surface of the cortex were set as two boundary lines of the potential field, and the potential field model $\psi$ was constructed. The potential field was described by a second-order partial differential equation, also known as the Laplace equation, as shown in Eq. (1).
  1 $$ \nabla^{2} \psi { = }\frac{{\partial^{2} \psi }}{{\partial x^{2} }} + \frac{{\partial^{2} \psi }}{{\partial y^{2} }} + \frac{{\partial^{2} \psi }}{{\partial z^{2} }} = 0 $$
  where the GM-CSF surface is the outer surface and the potential field value of the area outside the outer surface is set to 256. The WM-GM surface is the inner surface, and the WM area within the inner surface is set to a potential field value of 0.
  Then, we used the iterative method to solve the equipotential surface between the inner and outer surfaces of the 3D brain space, as shown in Eq. (2). The equipotential surfaces do not intersect each other and transform smoothly between the inner and outer surfaces.
  2 $$ \begin{aligned} \psi_{i + 1} (x,y,z) = & (\psi_{i} (x + \Delta x,y,z) + \psi_{i} (x - \Delta x,y,z) + \psi_{i} (x,y + \Delta y,z) \\ & + \psi_{i} (x,y - \Delta y,z){ + }\psi_{i} (x,y,z + \Delta z){ + }\psi_{i} (x,y,z - \Delta z))/6 \\ \end{aligned} $$
  where $\psi_{i} (x,y,z)$ potential energy value at position $(x,y,z)$ of the $i{\text{ - th}}$ iteration, $\Delta x = 1$, and $\varepsilon_{i} = {\text{sqrt}}[(\psi_{i + 1} - \psi_{i} )^{2} ]$. When $\varepsilon_{i} < 0.1$, the iteration is stopped, and the equipotential surface is obtained.
  Step 2: the field line was calculated according to the equipotential surface, as shown in Eq. (3). The field line is located between the inner and outer surfaces and perpendicular to all the equipotential surfaces.
  3 $$ \left\{ \begin{aligned} \left\{ {\Delta \psi (x,y,z)/\Delta x = } \right.[\psi (x + \Delta x,y,z) - \psi (x - \Delta x,y,z)]/2 \hfill \\ \left\{ {\Delta \psi (x,y,z)/\Delta y = } \right.[\psi (x,y + \Delta y,z) - \psi (x,y - \Delta y,z)]/2 \hfill \\ \left\{ {\Delta \psi (x,y,z)/\Delta z = } \right.[\psi (x,y,z + \Delta z) - \psi (x,y,z - \Delta z)]/2 \hfill \\ \end{aligned} \right. $$
  Then, the field line was normalized according to Eq. (4) of the gradient and the unit tangent vector of the point along the field line direction was obtained.
  4 $$ \left\{ \begin{aligned} \overrightarrow {T}_{x} = (\Delta \psi /\Delta x)/[(\Delta \psi /\Delta x)^{2} + (\Delta \psi /\Delta y)^{2} + (\Delta \psi /\Delta z)^{2} ] \hfill \\ \overrightarrow {T}_{y} = (\Delta \psi /\Delta y)/[(\Delta \psi /\Delta x)^{2} + (\Delta \psi /\Delta y)^{2} + (\Delta \psi /\Delta z)^{2} ] \hfill \\ \overrightarrow {T}_{z} = (\Delta \psi /\Delta z)/[(\Delta \psi /\Delta x)^{2} + (\Delta \psi /\Delta y)^{2} + (\Delta \psi /\Delta z)^{2} ] \hfill \\ \end{aligned} \right. $$
  Step 3: we used field line tracing to calculate the thickness of the cortex. The thickness of any point in the cortex can be defined as the length of the field line passing the point. We first calculated the number of steps from each point in the cortex to the GM-CSF boundary. For example, starting from a point $(x,y,z)$ on the boundary of GM-WM, set the step size to 0.1, and obtain the next point $(x + \Delta x,y + \Delta y,z + \Delta z)$ along the direction of the unit tangential vector $T(x,y,z)$, where $\Delta x = stepsize \cdot \overrightarrow {T}_{x} (x,y,z)$, $\Delta y = stepsize \cdot \overrightarrow {T}_{y} (x,y,z)$, and $\Delta z = stepsize \cdot \overrightarrow {T}_{z} (x,y,z)$. Then, we repeated the above operations to obtain the number of steps from each voxel to the GM-CSF boundary. As $\Delta x$, $\Delta y$, $\Delta z$ cannot always be an integer, the direction of the next point $T(x + \Delta x,y + \Delta y,z + \Delta z)$ has to be interpolated. The number of steps from each point in the cortex to the GM-WM boundary can be calculated in the same way and in the opposite direction $- T(x,y,z)$. Finally, the steps to the inner and outer boundaries were added to obtain the total steps for each voxel in the cortex. The true thickness is the total steps multiplied by the step size and multiplied by the resolution.
4. Convolution: the cortical thickness obtained in step (3) was smoothed by performing a 3D convolution with a matrix (convolution kernel) of 4^3^. This can reduce the high-frequency noise in the cortical thickness measurement, improve the signal-to-noise ratio of the image, make the thickness values closer to a normal distribution, and ultimately improve the detection accuracy.
5. Comparison with healthy controls: to find FCD lesion areas with different cortical thicknesses from epilepsy patients using HCs, a cortical thickness extension map, or z-score map, was computed by subtracting the cortical thickness mean image of the HCs from the smooth cortical thickness image of each individual and dividing the result by the cortical thickness standard deviation (SD) image of the HCs.
  The cortical thickness mean image and the cortical thickness SD image were calculated from the T1 images of 32 normal subjects. Each individual was processed from step (1) to step (4). The average of all individuals was taken to obtain the cortical thickness mean image, and the standard deviation of all individuals was taken to obtain the cortical thickness SD image.
6. Quantitative evaluation: after detection, the detected lesions and the manually segmented lesions were compared to qualitatively and quantitatively evaluate the detection effect. Lesion segmentation was performed in a semi-automatized and retrospective manner on the normalized brain images by an experienced epilepsy specialist depending on the surgical resection area. For the quantitative evaluation, we performed patient-wise and voxel-wise analyses. We used specificity, accuracy, recall, precision, Dice coefficient, Youden index, and area under the receiver operating characteristic (ROC) curve (AUC) for the quantitative evaluation [37]. The following equations were used: ${\text{Specificity}} = {\text{TN}}/({\text{TN}} + {\text{FP}}) \cdot 100$, ${\text{Accuracy}} = ({\text{TP}} + {\text{TN}})/({\text{TP}} + {\text{FP}} + {\text{FN}} + {\text{TN}}) \cdot 100$, ${\text{Recall = (}}{{\text{TP}} \mathord{\left/ {\vphantom {{\text{TP}} {{\text{TP}} + {\text{FN}}}}} \right. \kern-0pt} {{\text{TP}} + {\text{FN}}}} )\cdot 1 0 0$, ${\text{Precision}} = {\text{TP}}/({\text{TP}} + {\text{FP}}) \cdot 100$, ${\text{Dice coefficient}} = 2 \times \frac{{{\text{Precision}} \times {\text{Recall}}}}{{{\text{Precision}} + {\text{Recall}}}} \cdot 100$, ${\text{Youden index}} = {\text{Recall}} + {\text{Specificity}} - 1$, and ${\text{AUC}} = 1 - \frac{1}{2}\left( {\frac{\text{FP}}{{{\text{FP}} + {\text{TN}}}} + \frac{\text{FN}}{{{\text{FN}} + {\text{TP}}}}} \right),$
  where ${\text{TP}}$ are the actual lesion voxels that were identified; ${\text{FP}}$ are the healthy voxels incorrectly identified as lesion voxels; ${\text{FN}}$ are the actual lesion voxels incorrectly identified as healthy voxels; and ${\text{TN}}$ are the healthy voxels correctly identified as healthy voxels.

## Data Availability

The datasets during the current study are not publicly available due to some research that has not been completed, but is available from the corresponding author on reasonable request.

## Abbreviations

FCD: Focal cortical dysplasia
MR: Magnetic resonance
3-D: Three-dimensional
SD: Standard deviation
FLAIR: Fluid-attenuated inversion recovery
GM/WM: Gray/white matter
MCD: Malformation of cortical development
VBM: Voxel-based method
SBM: Surface-based method
PBT: Projection-based thickness
VBCT: Voxel-based cortical thickness
ARCTIC: Automatic regional cortical thickness
RLC: Run-length coding
GM-CSF: Cerebrospinal fluid
ECoG: Electroencephalogram
CNN: Convolutional neural network
T1WI: T1-weighted imaging
T2WI: T2-weighted imaging
MAP: Morphometric analysis programme
HC: Healthy controls
ROC: Receiver operating characteristic
AUC: Area under ROC curve
MSP: Mid-sagittal plane
