# Correction to: Detecting focal cortical dysplasia lesions from FLAIR-negative images based on cortical thickness

**DOI:** 10.1186/s12938-020-00760-9

**Published:** 2020-03-17

**Authors:** Cuixia Feng, Hulin Zhao, Maoyu Tian, Miaomiao Lu, Junhai Wen

**Affiliations:** 1 Department of Biomedical Engineering, School of Life Science, Beijing Institute of Technology, Beijing, China; 2 Sixth Medical Center of PLA General Hospital, Beijing, China

## Correction to: BioMed Eng OnLine (2020) 19:13 10.1186/s12938-020-0757-8

It was highlighted that the original article [1] contained an error in the Quantitative evaluation of Methods. A bracket was misplaced in the formula. This Correction article shows the incorrect and correct formula.

- **Incorrect formula**
- $$ {\text{Recall}} = \left( {{{\text{TP}} \mathord{\left/ {\vphantom {{\text{TP}} {{\text{TP}} + {\text{FN}}}}} \right. \kern-0pt} {{\text{TP}} + {\text{FN}}}}} \right) \cdot 100 $$
- **Correct formula**
- $$ {\text{Recall}} = {{\text{TP}} \mathord{\left/ {\vphantom {{\text{TP}} {\left( {{\text{TP}} + {\text{FN}}} \right)}}} \right. \kern-0pt} {\left( {{\text{TP}} + {\text{FN}}} \right)}} \cdot 100 $$
